# Advances of injectable hydrogel-based scaffolds for cartilage regeneration

**DOI:** 10.1093/rb/rbz022

**Published:** 2019-05-25

**Authors:** Jiawei Li, Guojun Chen, Xingquan Xu, Peter Abdou, Qing Jiang, Dongquan Shi, Zhen Gu

**Affiliations:** 1 Department of Sports Medicine and Adult Reconstructive Surgery, Drum Tower Hospital, School of Medicine, Nanjing University, 321 Zhongshan Road, Nanjing, Jiangsu, P.R. China; 2 Department of Bioengineering, University of California, Los Angeles, 410 Westwood Plaza, Los Angeles, CA, USA; 3 California NanoSystems Institute, University of California, Los Angeles, 570 Westwood Plaza, Los Angeles, CA, USA; 4 Jonsson Comprehensive Cancer Center, University of California, Los Angeles, 8-684 Factor Building, Los Angeles, CA, USA; 5 Center for Minimally Invasive Therapeutics, University of California, Los Angeles, 570 Westwood Plaza, Los Angeles, CA, USA

**Keywords:** drug delivery, tissue engineering, injectable hydrogel, cartilage regeneration

## Abstract

Articular cartilage is an important load-bearing tissue distributed on the surface of diarthrodial joints. Due to its avascular, aneural and non-lymphatic features, cartilage has limited self-regenerative properties. To date, the utilization of biomaterials to aid in cartilage regeneration, especially through the use of injectable scaffolds, has attracted considerable attention. Various materials, therapeutics and fabrication approaches have emerged with a focus on manipulating the cartilage microenvironment to induce the formation of cartilaginous structures that have similar properties to the native tissues. In particular, the design and fabrication of injectable hydrogel-based scaffolds have advanced in recent years with the aim of enhancing its therapeutic efficacy and improving its ease of administration. This review summarizes recent progress in these efforts, including the structural improvement of scaffolds, network cross-linking techniques and strategies for controlled release, which present new opportunities for the development of injectable scaffolds for cartilage regeneration.

## Introduction

Articular cartilage is a highly organized tissue which has remarkable load-bearing and low friction properties that allow for smooth movement of diarthrodial joints [1, 2]. The cartilage component contains sparsely distributed chondrocytes which are embedded within the extracellular matrix (ECM), mainly comprised water, type II collagen and glycosaminoglycans that provide the tissue with sufficient mechanical properties for several biofunctions, such as load-bearing and low friction capabilities [3, 4]. Due to the avascular, aneural and non-lymphatic characteristics of cartilage, cartilage has limitations in its self-regeneration and intrinsic repair [3]. Thus, cartilage regeneration still remains a challenge in tissue engineering [1].

Currently, strategies of repairing cartilage defects include debridement and lavage, microfracture, as well as autografts (cell and tissue transplantation) [5–7]. Although these therapies have exhibited some efficacy in the repair of cartilage defects, there are still certain limitations, such as poor integration with healthy cartilage, lack of nutrients, and the formation of fibrous tissue instead of hyaline cartilage that has a consistent morphology and function in clinical applications [8]. Typically, the inadequately regenerated cartilage does not have normal mechanical properties and zonal organization, which could most likely result in further degeneration [9]. The limitations of current therapies for cartilage regeneration have hence led to cartilage tissue engineering, which aims to combine engineering with biological principles to induce the regeneration of cartilage and to treat osteoarthritis [10–12].

**Figure 1.**
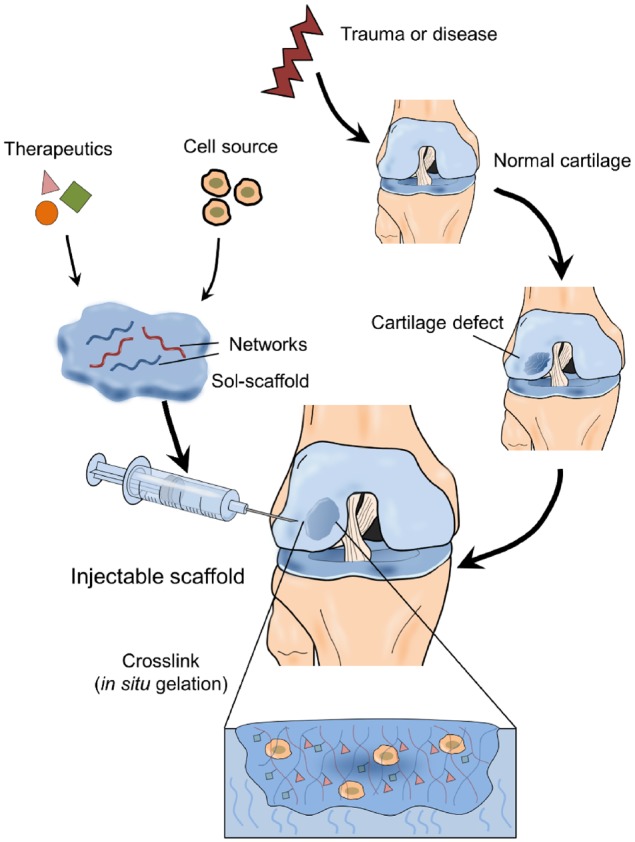
The schematic of the applications of injectable scaffolds for cartilage regeneration

To further expand the utilization of biomaterials in cartilage regeneration, injectable hydrogel-based scaffolds have attracted considerable attention these years in cartilage tissue engineering [13, 14]. Hydrogels are notably swollen and porous with 3D polymeric networks, where various solutes and nutrients can be located and able to diffuse [15–18]. Furthermore, as illustrated in Fig. 1, injectable scaffolds can be delivered in a non-invasive or minimally invasive manner via either direct injection or arthroscopy. Injectable hydrogels can not only provide a biocompatible, biodegradable and highly hydrated 3D structure analogous to cartilaginous ECM and improve the supply of nutrients and cellular metabolites via elastic properties [19–22], but also encapsulate cells and deliver bioactive molecules efficiently and effectively through stimuli-responsive release mechanisms to targeted sites for cartilage regeneration [13, 23–26].

An ideal injectable scaffold for cartilage regeneration should typically meet the following criteria: (i) ease of administration under physiological conditions, (ii) guaranteed injectability (gelation upon injection via either chemical or physical cross-linking), (iii) excellent biocompatibility and potential biodegradability, (iv) the ability to mimic cartilaginous ECM features and promote chondrogenic potential of cells, (v) the ability to easily fill defect sites inside the joint and integrate with the surrounding native cartilage tissue rather than shifting readily and (vi) a sustained release profile if associated with local drug delivery [13, 15, 27–29]. This review aims to provide an overview of the current advances of injectable scaffolds in cartilage regeneration with an emphasis on the components of scaffolds and cell sources.

## The structure of injectable scaffolds

Hydrogels possess high water content and elastic properties with cross-linked, multiporous networks [30]. The potential of hydrogels as efficient biomaterials have been reported since the latter half of the 20th century, beginning with the use of nondegradable methacrylate gels to fabricate soft contact lenses [31, 32]. Thereafter, people have investigated hydrogels for various biomedical applications, including drug delivery, wound healing and tissue engineering [33]. Hydrogels can be broadly classified based on the source material (natural or synthetic) and biodegradability (biodegradable or non-biodegradable). Natural hydrophilic macromolecules used for hydrogel scaffold fabrication are often biodegradable and mainly consist of proteins and polysaccharides [34]. Natural polysaccharides used to prepare injectable hydrogels for tissue engineering include chitosan (CS), alginate, agarose and hyaluronic acid (HA) [35]. Protein-based materials, such as collagen, gelatin and fibrin, are also popular for engineering bioactive scaffolds because of their advantages in mimicking the extracellular environment [36–39]. Hydrogels derived from synthetic polymers are more chemically programmable and tunable to systematically determine their cell–matrix interactions [30]. Some examples of synthetic polymers that have been utilized in cartilage regeneration engineering include poly(ethylene glycol) (PEG), poly(vinyl alcohol) (PVA), polydioxanone as well as poly(lactic acid) [23]. For example, PEG has been extensively investigated for biomedical applications because of its good tissue compatibility, nontoxicity and hydrophilicity [30]. Notably, as aforementioned, because biodegradability is an essential characteristic of injectable scaffolds for cartilage tissue engineering, several degradable PEG-based synthetic polymeric systems have also been developed to form hydrogels, including copolymers of PEG with a diverse array of synthetic degradable polymers, such as poly(propylene fumarate), poly(lactic-co-glycolic) acid (PLGA), PVA, polyanhydrides, poly(propylene oxide) and polyphosphazenes [40]. In cartilage tissue engineering, these biomimetic polymers, either natural or synthetic, are designed to mimic crucial aspects of the native extracellular environment by distinctly adjusting mechanical, chemical and biological properties of hydrogels [41].

### Multilayer structure of injectable scaffolds

**Figure 2.**
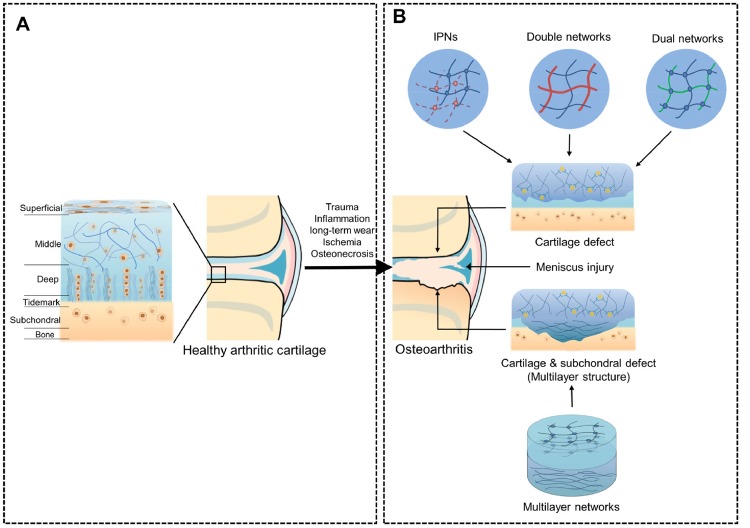
(**A**) The schematic of the anatomy, cell morphology and zonal features of articular cartilage, and its progression to different types of osteoarthritis. (**B**) The schematic of different structure scaffold networks utilized in the cartilage regeneration engineering

**Figure 3.**
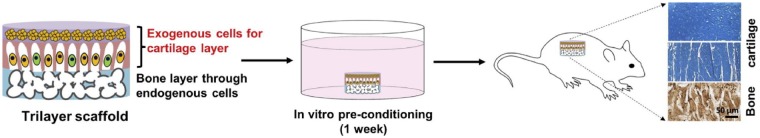
The implantation of the cell-laden trilayer scaffold resulted in the formation of osteochondral tissue with a lubricin-rich cartilage surface. This figure was adapted with permission from Kang *et al.* [47]

Articular cartilage in joints is divided into the superficial, middle, deep and calcified zones (Fig. 2A). These zones have different cell morphologies, compositions, structural arrangements of the ECM and mechanical properties [42]. Clinically, the most symptomatic cartilage damage is the osteochondral injury with involvement of both the cartilage and subchondral layers. To simulate the complex zonal architecture of cartilage and the zones likely damaged by osteochondral defects in the joint, as shown in Fig. 2B, multilayer injectable hydrogels attract particular interest for cartilage tissue engineering. In the early stages of work toward this goal, a bilayer hydrogel system was developed, which constituted a simple form of a multilayer matrix [43]. Cui *et al*. [44] and Sun *et al.* [45] investigated bilayer hydrogels by both 3D printing and projection of stereolithography. Nguyen *et al.* [46] designed a PEG-based tri-layer hydrogel with the first layer comprising chondroitin sulfate (CHS) and matrix metalloproteinase-sensitive peptides, the second layer comprising CHS and PEG, and the third layer comprising PEG and HA. It was demonstrated that this complex construct could encapsulate a single line of stem cells in all layers and could increase the production of type X collagen and proteoglycans. Kang *et al.* [47] fabricated a single-unit tri-layer scaffold to engineer osteochondral tissues *in vivo*, including a biomineralized bottom layer mimicking a calcium phosphate (CaP)-rich bone microenvironment, a middle hydrogel layer with anisotropic pore structure and a top hydrogel layer (Fig. 3). These tri-layer scaffolds contributed to the regeneration of osteochondral tissue with a lubricin-rich cartilage surface, which was similar to the native tissue. Furthermore, this scaffold significantly enhanced the sustained differentiation of transplanted cells to form neocartilage tissue and the recruitment of the surrounding endogenous cells to form bone tissues through the bottom layer. In addition to the osteochondral tissue repair, theoretically, it is also ineffective for monolayer scaffolds to be used for cartilage regeneration due to the complex hierarchical structure of cartilage. It remains to be seen how advances in the design of hydrogels will impact their ability to mimic the structure, properties, and arrangement of cells and collagen fibers of the native ECM.

### Interpenetrating polymer network of scaffolds

Mechanical integrity is a crucial design criterion for hydrogels in cartilage regeneration. However, networks of traditional hydrogels are generally based on a single polymer, resulting in reduced mechanical properties, which are inferior to those of natural cartilage [10]. To enhance the mechanical properties of hydrogels to better mimic natural hyaline cartilage, as shown in Fig. 2B, the research focus is transitioning from conventional hydrogels that consist of a single polymer to the hydrogel systems integrated with two or more independent networks with superior functions [48, 49].

Interpenetrating polymer network (IPN) hydrogels were developed aiming to enhance its mechanical properties [50]. IPN, a type of unique mixture of polymers, is composed of two or more cross-linked networks, which are partially intertwined with each other rather than covalently linked [51]. According to recent reports, hydrogels with IPNs tend to exhibit superior mechanical properties compared with those formed by a single type of polymer. Snyder *et al.* [52] demonstrated that an IPN of cross-linked HA improved the mechanical strength of hydrogel constructs and increased the expression of the chondrogenic transcription factor Sox9 by the loaded human mesenchymal stem cells (MSCs). Gan *et al.* [50] investigated the incorporation of a primary network consisting of dextran and gelatin with a PEG-based secondary network. This IPN hydrogel showed high toughness and increased proliferation, clustering and matrix deposition of encapsulated nucleus pulposus cells when the quantity of the primary network was 4-fold greater than the secondary one. Chen *et al.* [51] investigated the combination of a sodium hyaluronate/sodium alginate (HA/SA)-based scaffold with berberine and found that this system could stimulate the regeneration of cartilage as well as subchondral bone. Furthermore, Guo *et al.* [53] investigated a tri-component IPN hydrogel (consisting of collagen, methacrylate-modified CHS and HA), which can better mimic the natural materials in articular cartilage.

Furthermore, double IPN networks and dual IPN networks have been investigated with the aim of developing injectable scaffolds. A double network consists of two polymers combined with different mechanical properties (rigid versus ductile), which leads to a hydrogel matrix with greater toughness than the corresponding single polymer network alone [54]. Therefore, double networks have attracted much interest in cartilage tissue engineering. A study comparing double-network hydrogels and traditional single-network hydrogels of either poly(2‐acrylamido‐2‐methylpropanesulfonic acid) (PAMPS) or poly(*N*,*N*′‐dimethyl acrylamide) (PDMAAm) demonstrated that the double-network hydrogel structure showed superior cartilage regeneration by histological and biochemical scoring [55]. Stagnaro *et al.* [56] built a porous scaffold based on alginate–polymethacrylate hybrid hydrogels and demonstrated that this matrix mimicked the microenvironment of the ECM in cartilage tissue by overcoming mechanical limitations. Levett *et al.* [57] generated a double-network hydrogel formed by gelatin and HA. Due to the high reactivity of methacrylate groups, this hydrogel matrix system exhibited advantages in terms of the compressive modulus and chondrification by encapsulating human chondrocytes [57]. In contrast to the double networks, the dual networks consist of two materials with similar cross-linking mechanisms. However, these two similar components have different and mutually beneficial properties. For example, a dual-network hydrogel consisting of HA with a high molecular weight (>1600 kDa) with PVA with a low molecular weight (27 kDa) was constructed by Pirinen *et al.* [58], which were further chemically cross-linked by the reaction between aldehydes and primary amines. The swelling properties can be tuned by varying the size of the PVA component. Enhanced cell viability of encapsulated bovine knee chondrocytes was observed with the addition of HA [58].

#### Nanocomposites integrated in scaffolds

In bone or cartilage tissue engineering, the load-bearing property of the material is a crucial feature. The stiffness of hydrogel scaffolds is 2 orders of magnitude lower than natural cartilage [59]. The high water content of hydrogels and their limited stiffness are the two main drawbacks of the progression of cartilage regeneration *in vitro* and *in vivo* [60]. Researchers have reported that these hindrances could be alleviated if nanomaterials were added into hydrogel scaffolds [61]. Thus, nanocomposite hydrogel systems have attracted increasing attention in recent years.

Hybrid hydrogels integrated with nanoscale composites are defined as hydrated polymeric networks which are either physically or chemically cross-linked with nanoparticles (NPs) or other nanostructures [62]. Nanoparticles can act as fillers to improve the mechanical properties of hydrogel scaffolds [63]. Different types of NPs, including carbon-based nanomaterials (such as carbon nanotubes, graphene and nanodiamonds), inorganic/ceramic NPs (such as hydroxyapatite, silica, silicates and calcium phosphate), polymeric NPs and metal/metal oxide NPs (such as gold, silver and iron oxide), can be incorporated in the polymeric network to form nanocomposite hydrogels [62]. Nanoscale composites with large surface area-to-volume ratios can not only improve the surface reactivity but also provide enhanced mechanical properties. In addition, because they can easily penetrate into the focal tissue via narrow or small capillaries or the epithelial lining, the efficacy of loaded therapeutic agents and bioactive agents can be enhanced [64–66].

Several nanomaterials have been developed as injectable scaffolds to mimic the ECM of cartilage. For example, Zhang *et al.* [67] synthesized a hybrid hydrogel (MagGel), composed of type II collagen, HA, PEG and magnetic NPs for cartilage regeneration. The MagGel showed similar microstructure and chemistry as hyaline cartilage and was cytocompatible with bone marrow mesenchymal stem cells (BMSCs) *in vitro*. Interestingly, MagGel could be used to direct the scaffold remotely to the cartilage defect site using an external magnet [67]. Radhakrishnan *et al*. [68] investigated a semi-interpenetrating network hydrogel scaffold formed by CHS NPs and nanohydroxyapatite used in chondral and subchondral hydrogel layers, respectively. The regeneration of subchondral bone and cartilage tissue was enhanced by this hybrid hydrogel [68]. Boyer *et al.* [69] developed a hybrid interpenetrating network mixed with laponites, known as a nano-reinforcing clay, and silated hydroxylpropylmethyl cellulose, which increased the hydrogel mechanical properties without compromising its oxygen diffusion capability, cytocompatibility, the self-organization of chondrogenic cells and generation of ECM components. Collectively, the functions of the added nanocomposite are as follows: (i) mechanical reinforcement, (ii) biological activity and biomimetic function, (iii) integration of cartilage with bone tissue and (iv) transport of drugs and growth factors [70, 71].

## Formation of scaffolds

To guarantee the injectability of scaffolds, the scaffolds are typically in a solution state before administration and proceed to an *in situ* gelation after administration. Furthermore, while developing an ideal injectable hydrogel scaffold that can undergo *in situ* gelation for better usage in cartilage tissue engineering, it should also ideally meet the following criteria: (i) solubility in aqueous media with gelation occurring under physiological changes (such as temperature, pH and ionic concentration), (ii) no release of harmful byproducts during gelation and (iii) a suitable rate of gelation for practical use [30]. The process of hydrophilic polymers undergoing *in situ* gelation in response to various stimuli has been developed by either tuning the components of polymers or specifying the sensitive units in polymer chains. As shown in Table 1, the stimulation of gelation applied for cartilage tissue engineering includes functions of chemical agents, physiological stimuli and light.

**Table 1.** Advances in formation of injectable scaffolds for cartilage regeneration

	Formation of hydrogels	Major materials
Physically cross-linked hydrogels	Thermosensitive	Pluronics [72]
		P(NIPAAm) [74]
		PLGA-PEG-PLGA [76]
		CS/GP [88–91]
	Thermosensitive	Gelatin-Pluronic copolymer [79]
		CS/hydroxyethyl cellulose [81]
		CS/HA [83]
	pH-responsive	CHS–PEG [94]
		Poly(methacrylic acid) [95]
	Ion-responsive	Hyaluronate-*g*-alginate [96]
Chemically cross-linked hydrogels	Schiff base reaction	CS/aldehyde HA [99]
		Glycol CS/poly(EO-*co*-Gly)-CHO [100]
	Click Chemistry	Azadibenzocyclooctyne-modified and azide-modified Dextran [104]
	Michael addition reaction	Amino derivative of HA/divinylsulfone [110]
	Enzyme-catalyzed cross-linking	Heparin-tyramine/dextran-tyramine/HRP [115]
	Photo-cross-linking	Poly(ethylene glycol)dimethacrylate/UV [117]
		Sericin methacryloyl/UV [118]
		Methacrylated glycol CS and HA/Visible light [116]

### Physically cross-linked hydrogels

Thermosensitive hydrogels are one of the most extensively studied injectable hydrogel systems for tissue engineering. The sol–gel transition occurs either above or below a critical temperature, termed as lower critical solution temperature or upper critical solution temperature. The most commonly used thermogels in cartilage tissue engineering include Pluronics [72], p(NIPAAm) [73, 74], poly(*N*-vinylcaprolactam) [75] and PLGA–PEG–PLGA [76–78]. For instance, Li *et al.* [76] loaded kartogenin (KGN) into PLGA–PEG–PLGA thermogel as an injectable scaffold for BMSCs, which exhibited good mechanical properties and effective cartilage regeneration *in vivo*. Moreover, these polymers are often mixed or conjugated with other natural polymers, such as gelatin [79, 80], cellulose [81] and HA [82, 83] to improve the biocompatibility and mechanical properties of the thermogels. [75, 84]. For example, Lynch *et al.* reported poly(*N*-vinylcaprolactam)-graft-HA with a lower critical solution temperature of around 33°C. This injectable thermosensitive hydrogel improved cell compatibility and promoted the generation of ECM proteins even under hypoxic conditions [75]. CS has attracted great attention as an injectable hydrogel scaffold for cartilage repair because of its structural similarity to glycosaminoglycan [81, 85, 86]. Formation of CS-based thermosensitive hydrogels can be achieved by mixing with *β*-glycerophosphate (GP), which can increase the pH of the CS solution to a range of 7.0–7.4 and allow for gel formation at body temperature [87]. The CS/GP thermogel has been widely applied to cartilage regeneration [88–91]. The mixture of CS with other polymers has also been investigated [92, 93]. For example, Qi *et al.* [92] designed a CS/PVA-based thermoresponsive hydrogel combined with rabbit BMSCs transfected with hTGF*β*-1 to repair rabbit articular cartilage defects. The non-degradable PVA can postpone the degradation of the CS/PVA gel, thereby prolonging the self-repair duration. pH-responsive injectable scaffolds have also been investigated for cartilage tissue engineering. The formation of pH-responsive hydrogels mostly engages dissociation and association with H^+^ in response to the changes of environmental pH. This type of hydrogel is studied extensively for biomedical applications because the pH profiles at pathological tissues (such as inflammation, infection and cancer) differentiate from that of normal tissues. Strehin *et al.* [94] developed pH-responsive CHS–PEG adhesive hydrogels with potential applications in regenerative medicine including cartilage repair. The stiffness, swelling properties and gelation kinetics of the hydrogel can be tuned by adjusting the initial pH values of the precursor solutions. Halacheva *et al.* [95] reported a poly(methyl methacrylate-*co*-methacrylic acid)-based pH-sensitive hydrogel with high porosity, elasticity and ductility. The enhanced mechanical properties of the injectable hydrogel make it a good candidate for regenerative medicine.

Ion-sensitive injectable hydrogels have also been developed for cartilage regeneration. The hyaluronate-*g*-alginate solution can easily form a hydrogel by adding Ca^2+^ [96]. This ionically cross-linkable hydrogel provided an appropriate scaffold to transplant chondrocytes, resulting in efficient chondrogenic differentiation in cartilage regeneration.

### Chemically cross-linked hydrogels

Chemically cross-linked hydrogels have been extensively utilized for tissue engineering [30]. Versatile chemistry enables the integration of functional groups into the polymers, allowing for *in situ* cross-linking. A variety of chemical reactions have been investigated to generate injectable hydrogels for cartilage regeneration, including Schiff base reaction, click chemistry, Michael addition, enzyme-catalyzed cross-linking and photo-cross-linking.

#### Gel formation by Schiff base reaction

Injectable hydrogels formed by Schiff base reaction between amine and carbonyl groups have been widely utilized for cartilage regeneration applications, owing to the high reaction rate, mild reaction conditions as well as good biocompatibility [97, 98]. CS, carrying abundant amino groups, is an excellent polymer candidate for synthesizing injectable hydrogels through Schiff base cross-linking. For example, Tan *et al.* [99] reported an injectable CS–HA hydrogel via Schiff base reaction for potential cartilage tissue engineering. Gelation time, degradation profile and mechanical properties can be controlled by adjusting S-CS/A-HA ratios. Cao *et al.* [100] designed a chemically cross-linked hydrogel via Schiff base reaction using glycol CS and aldehyde-functionalized PEG (poly(EO-co-Gly)-CHO) with a flexible capability to tune the properties of hydrogels.

#### Gel formation by click chemistry

Click chemistry involves a wide range of reactions, such as azide–alkyne cyclo-addition reactions, thiol–ene couplings, Diels–Alder reactions and tetrazine–norbornene chemistry [101]. These reactions have been widely used to fabricate injectable hydrogels, owing to their rapid reaction kinetics and low reactivity with cellular components [102, 103]. Wang *et al.* [104] reported dextran-based hydrogels formed by metal-free biorthogonal click chemistry. They used non-toxic metal-free azide–alkyne addition to form injectable hydrogels, which made it applicable for *in vivo* use. Yu *et al.* [105] prepared an *in situ* formed HA/PEG hydrogel via a two-step cross-linking method. The first step was the enzymatic cross-linking between tyramine groups of furylamine-grafted HA and the second step was the Diels–Alder click chemistry between unreacted furan-modified HA and dimaleimide PEG. The two-step cross-linking showed improved mechanical properties of the hydrogel.

#### Gel formation by Michael addition reaction

The Michael addition reaction has been commonly used to prepare injectable hydrogels, ascribed to its mild reaction condition and controllable reaction time [106–108]. Jin *et al.* [109] reported on an injectable hydrogel based on thiolated HA and PEG vinylsulfone via Michael addition. The gelation and degradation time can be adjusted by varying polymer concentrations and molecular weights of polymers. Fiorica *et al.* [110] prepared two kinds of HA-based injectable hydrogels by Michael addition, using the amino derivative of HA (HA-EDA), α-elastin-grafted HA-EDA and *α,β*-poly(*N*-2-hydroxyethyl)-dl-aspartamide derivatized with divinylsulfone. The controllable swelling and degradation kinetics and its ability to integrate articular chondrocytes of the hydrogel suggested that this scaffold processed desirable properties for cartilage regeneration.

#### Gel formation by enzyme-catalyzed cross-linking

The enzyme-catalyzed chemical cross-linking method has attracted increasing attention for hydrogelation, due to its fast gelation rate, high site specificity, ability to work at normal physiological conditions and low cytotoxicity [111]. There have been many attempts to produce enzymatically cross-linked hydrogels for cartilage tissue engineering, including transglutaminase, tyrosinase, phosphopantetheinyl transferase, lysyl oxidase, plasma amine oxidase and horseradish peroxidase (HRP) [112–114]. Particularly, Teixeira *et al.* showed that the HRP-mediated cross-linking systems can covalently bind the phenol-conjugated polymers (heparin-tyramine and dextran-tyramine conjugates) to the ECM proteins of the surrounding tissues, which is beneficial in maintaining the structural integrity for arthroscopic cartilage repair [115].

#### Gel formation by photo-cross-linking

Photo-cross-linking involves multiple steps, including initiation, propagation and termination, under light illumination. This method requires the introduction of free radical groups, such as vinyl and methacrylate residues together with photo-initiators. In recent years, the photo-cross-linking method has been widely applied to synthesize injectable hydrogels for cartilage tissue engineering owing to the flexible ability to control the timing and location of hydrogel cross-linking [116]. For example, Papadopoulos *et al.* [117] reported a swine auricular chondrocyte encapsulated poly(ethylene glycol)dimethacrylate copolymer-based hydrogel by photo-cross-linking for cartilage repair. Neocartilage resembled both composition of ECM and cellular population of the native cartilage, indicating the promise for cartilage regeneration. Qi *et al.* [118] designed a sericin methacryloyl (SerMA)-based UV cross-linking hydrogel, which was adhesive to chondrocytes and promoted the proliferation of attached chondrocytes even in a nutrition-deficient condition. *In vivo* implantation of chondrocyte-loaded SerMA hydrogels adequately formed artificial cartilages. Although UV-mediated cross-linking is characterized by low cytotoxicity, UV radiation may still have a negative influence on cells, proteins and tissues. Hence, considerable attempts in visible-light-initiated polymerization for cartilage repair have been investigated. For instance, Park *et al.* [116] reported a visible light-induced photo-cross-linking of methacrylated glycol CS and HA hydrogels. Choi *et al.* [119] also reported the incorporation of cartilaginous ECM components into an injectable CS hydrogel designed to undergo gelation upon exposure to visible light.

## Incorporating cells into injectable scaffolds

Hydrogels are versatile and their various properties, such as high water content, biodegradability, porosity and biocompatibility, allow them to be used often for cell therapy [16, 120]. In cartilage tissue engineering, properly engineered hydrogel scaffolds are able to control cell proliferation and differentiation. Using advanced techniques, cell encapsulated hydrogels can also be fabricated with personalized geometries and compositions [30, 121, 122]. Over the last decade, various types of cell-loaded injectable hydrogel systems have been investigated for cartilage regeneration [30, 122]. Incorporation of cells into hydrogels can be realized by either seeding cells into the prefabricated porous scaffolds or encapsulating cells during scaffold formation. However, the cell lines that can be used for injectable scaffolds in cartilage regeneration are limited. Table 2 lists the examples of cells that have been incorporated in the injectable hydrogel scaffolds for cartilage regeneration [123–133].

**Table 2.** Examples of incorporation of cells into injectable scaffolds for cartilage regeneration

	Cell source	Major materials	Advantages
Chondrocytes (fully differentiated cells)	Chondrocytes	CS	Prolonged cell survival, retained cell morphology and improved chondrogenesis when cultured *in vitro* [137]
	Chondrocytes	CS and type II collagen	Improved cellular condensation and chondrogenesis of embedded chondrocytes to promote cartilage regeneration [136]
	Chondrocytes	Oligo(lactic acid)-*b*-PEG- *b*-oligo(lactic acid) (PEG-LA)	Improved formation of cartilage matrix of aggrecan and collagen type II/VI [138]
Stem cells	ESCs	PEG	Promoted ESC differentiation into chondrogenic cells and formation of neocartilage ECM [141]
	MCS	Agarose, hyaluronan acid, PEG or alginate	Increased chondrogenic differentiation of the cells for the cartilage reconstruction [50, 144–146]
	iPSCs	Polylactic	Prompted cartilage regeneration of an osteochondral defect [150]
	PBMCs	Graphene oxide (GO)-polyethylenimine (PEI)	Easily obtained from peripheral blood and have a similar potential of chondrogenic differentiation and cartilage generation compared with MSCs [151]

### Fully differentiated chondrocyte-encapsulated hydrogels

Autologous chondrocyte implantation has been successfully used in clinic to treat cartilage defects [5]. However, it is still challenging to directly fix a chondrocyte graft in a focal cartilage defect site with a complex shape by invasive orthopedic surgeries [123]. Therefore, injectable scaffolds have been proposed to overcome this challenge. Many reports indicate that chondrocytes can proliferate well in hydrogels and express cartilage-related proteins or genes with well-maintained cell morphologies and phenotypes [124, 134–136]. Jin *et al.* [137] developed an injectable CS-based hydrogel and found that it could support long-term chondrocyte survival and retain cell morphology *in vitro*. During *in vitro* culture, chondrogenesis occurred with the formation of cartilage ECM, including type II collagen and aggrecan, which were homogenously distributed throughout the entire hydrogel. Roberts *et al.* [138] demonstrated that a chondrocyte-laden hydrogel consisting of oligo(lactic acid)-*b*-PEG-*b*-oligo(lactic acid) improved the formation of a cartilage matrix consisting of aggrecan and collagen types II/VI. Although chondrocyte-based biomaterial therapy has demonstrated promising in cartilage tissue engineering, two notable limitations should be considered. First, chondrocyte harvesting involves collecting healthy cartilage tissues from non-load-bearing areas and long-term *in vitro* culturing (∼1 month) [139, 140]. Because of the low quantity of chondrocytes, and because cartilage defects cannot regenerate, the donor area can become necrotic using this approach. Second, autologous chondrocyte therapy is nearly ineffective in elderly patients due to the low bioactivity and proliferation capacity of autologous primary chondrocytes.

### Stem cells encapsulated in hydrogels

Biomedical therapies incorporating stem cells and hydrogels for cartilage regeneration commonly include ESCs, MSCs, induced pluripotent stem cells (iPSCs) and predifferentiated MSCs. ESCs, isolated from the tissues of early embryos, show an unlimited self-renewal capacity while maintaining a pluripotent differentiation potential [127]. However, stem cell pluripotency leads to difficult control over differentiation. Hwang *et al.* [141] have reported that combining these cells with biomimetic hydrogels and growth factors (such as transforming growth factor *β*1 and bone morphogenetic protein) created a synergistic environment for chondrogenesis. The encapsulated ESCs were able to differentiate into chondrogenic cells and promote the production of neocartilage ECM [141]. MSCs, derived from a variety of tissue sources, including bone marrow, adipose tissue, periodontal ligament, muscle, lung, liver, amnion, thymus, spleen, placenta, umbilical cord blood and corneal stroma, can interact with local biochemical stimuli to generate growth factors for multiple biofunctions for tissue regeneration [142, 143]. MSCs have become the most extensively used stem cells in biomedical applications due to their abundant cell sources, low immunogenicity, no ethical concerns and minimal teratoma risk [142]. Ample studies regarding the encapsulation of MSCs in chondrogenic 3D injectable hydrogels, such as agarose, hyaluronan acid, PEG and alginate, have been reported for the chondrogenic differentiation of cells and for the targeted reconstruction of cartilage [52, 144–147]. Notably, *in vitro* research has demonstrated that MSC proliferation and differentiation potential decreases with aging and with aging-related diseases [148], likely preventing their clinical applications in elderly individuals.

Recently, iPSCs have attracted significant attention because they exhibit pluripotency that is quite similar to ESCs in terms of multiple differentiation routes, thus resulting in increasingly widespread applications in regenerative medicine, which can be obtained from somatic cells including fibroblasts [129, 149]. Xu *et al.* [150] have demonstrated that human-derived iPSCs can maintain their pluripotency in a polylactic-based scaffold and are capable of regenerating cartilage in an osteochondral defect within 6 weeks in rabbits. Currently, it is feasible to produce iPSCs by using an integration-free approach with the development of cellular reprogramming techniques, which is safer and more amenable from a regulatory perspective for their future clinical applications [151]. Recently, predifferentiated MSCs, which can be easily obtained from peripheral blood with minimal invasiveness, have been reported to have a similar potential for chondrogenic differentiation and cartilage generation ability compared with MSCs [152]. However, their application potential in injectable scaffolds requires further study.

## Controlled-release drug delivery scaffolds

Many therapeutics exhibit limited efficacy due to the rapid clearance of the drugs in joints. Injectable scaffolds, on the other hand, can sustain drug release and extend the drug retention time. Numerous studies have investigated natural and synthetic biomaterials to develop scaffolds with unique properties, such as improved joint articular dwelling time with sustained drug release while ameliorating the biodegradation of delivery systems. Strategies investigated for the release of biologics with biological activity for treating cartilage defects have developed from simple bolus injections into the focal cartilage defect to multifunctional delivery systems.

**Figure 4.**
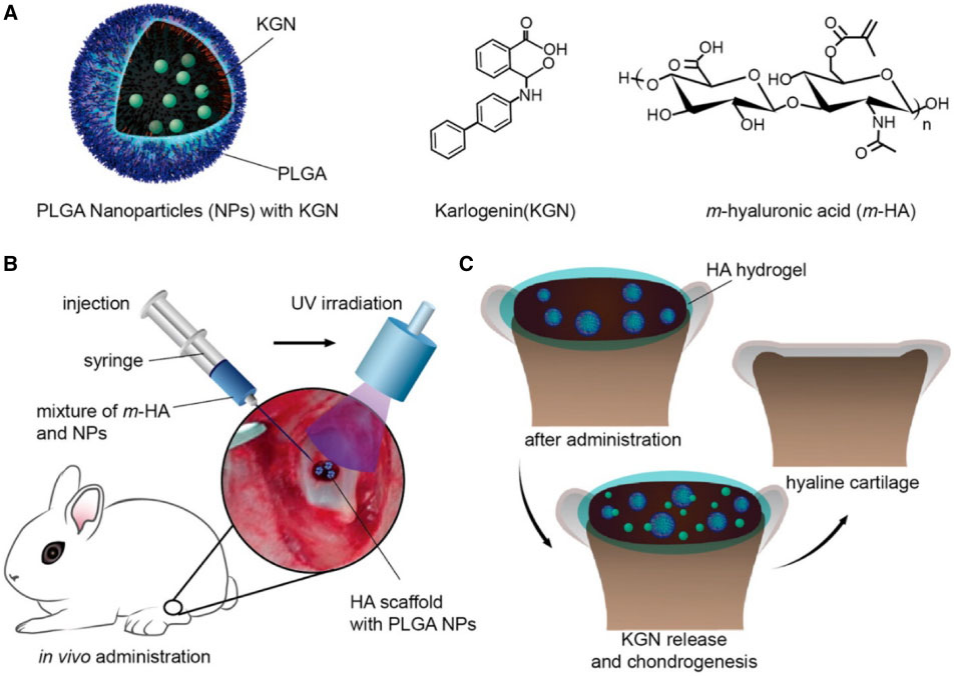
(**A**) Schematic of KGN-loaded PLGA NPs, molecule structures of KGN and acrylated HA (m-HA). (**B**) Schematic of the surgical procedure for cartilage defect repair. (**C**) Schematic of the hyaline cartilage chondrogenesis with the photo-cross-linked HA scaffold encapsulated with KGN-loaded NPs. This figure was adapted with permission from Shi *et al.* [161]

Microparticles (MPs) and NPs are desirable formulations for controlled drug release due to their high surface area to volume ratios, small dimensions, high drug encapsulating efficiencies and the capacity to quickly respond to surrounding environmental stimuli, such as temperature, pH, magnetic fields or ultrasound [153–155]. Recently, there have been many advancements in the application of MP and NP delivery vehicles for cartilage repair. One material that has received attention for the construction of MPs and NPs is the synthetic PLGA polymer, because of its controllable degradation profiles, ease of fabrication and safety in other FDA-approved applications [156]. Spiller *et al.* [157] have recently designed a hybrid scaffold system composed of PVA and PLGA loaded with insulin-like growth factor 1 (IGF-1). They used a double emulsion technique to form evenly dispersed PLGA MPs (11.3 ± 6.4 μm) containing IGF-1 throughout the PVA hydrogel, resulting in the release of IGF-1 in a linear and sustained manner for at least 45 days. Researchers have also designed NPs to deliver growth factors [158–160]. Recently, the Shi group reported that KGN can be encapsulated into biodegradable PLGA NPs through an emulsion-based formulation method, embedded in photocross-linked acrylated HA injectable hydrogels, and the release rate of KGN associated with the HA matrix integrated with KGN-NPs was nearly linear without an apparent burst release during the 2-month experimental period (Fig. 4) [161]. This injectable scaffold with a sustained release of KGN facilitated the filling of the defects and generation of hyaline cartilage. In another study, it has shown that NPs with a diameter of <10 nm can penetrate bovine cartilage explants, while NPs over 15 nm were limited to the superficial cartilage layer. Of note, a positive fixed charge density promoted the uptake of protein and enhanced protein retention to over 15 days, which was much longer compared with neutrally charged protein [162].

## Summary and future outlook

To date, injectable scaffolds have provided a promising therapeutic platform for cartilage regeneration. As surveyed above, a number of hydrogel-based scaffolds have been developed with inherent capabilities in cartilaginous tissue engineering, and sufficient mechanical properties for repairing cartilage defects to restore normal joint function. First, to enhance the mechanical properties of scaffolds, traditional single-network hydrogels have been supplemented with either additional networks or mixtures of polymers, and many nanocomposites have been utilized to vary the mechanical properties of scaffolds. These strategies have also been used to produce hydrogels which can improve the integration with surrounding cartilage while promoting chondrogenesis of stem cells encapsulated in hydrogels *in vivo*. Second, to enhance the efficiency and duration of the delivery of growth factors or other pharmaceuticals, advanced formulations such as MPs and NPs have been investigated in scaffolds for controlled drug delivery. These advances have also garnered interest in presenting biochemical cues in a controllable manner.

Looking ahead, there are still limitations of injectable scaffolds that restrict the complete regeneration of articular cartilage. First, it is essential that the injectable scaffolds can fill the defect area with a smooth interface that is similar to the native cartilage, without integrating into the surrounding healthy tissue. Second, the progressive degradation of hydrogels before they can be replaced by the *de novo* ECM could compromise their mechanical stability and long-term therapeutic efficacy. One option to overcome this issue is to incorporate appropriate exogenous cells, such as MSCs, within these scaffolds, which could potentially replace the scaffolds as they degrade with newly formed tissue. Third, signaling pathways and particular mechanisms from stem cells to specific cartilaginous cells need further in-depth understanding. It emphasizes more fundamental biological studies of cartilage development and regeneration, which could significantly contribute to the optimization of the injectable scaffolds in the long run. It is also essential to highlight the potential translation of the systems at the beginning of the design. Factors, such as biocompatibility of materials, ease of administration, feasibility of large-scale manufacturing and overall cost should be thoroughly evaluated. Lastly, the next generation of cartilage tissue engineering could be combined with noninvasive/minimally invasive diagnostic technologies to provide real-time assessment of the disease status and overall treatment performance, leading to personalized therapy.
